# Sofosbuvir-Based Therapy for Genotype 4 HCV Recurrence Post-Liver Transplant Treatment-Experienced Patients

**DOI:** 10.1155/2016/2872371

**Published:** 2016-04-11

**Authors:** A. Ajlan, A. Al-Jedai, H. Elsiesy, D. Alkortas, W. Al-Hamoudi, R. Alarieh, M. Al-Sebayel, D. Broering, F. Aba Alkhail

**Affiliations:** ^1^Pharmaceutical Care Division, King Faisal Specialist Hospital and Research Centre, MBC-11, P.O. Box 3354, Riyadh 11211, Saudi Arabia; ^2^Alfaisal University, College of Medicine, Riyadh, Saudi Arabia; ^3^Liver & Small Bowel Transplant and Hepatobiliary and Pancreatic Surgery-Organ Transplant Center, King Faisal Specialist Hospital & Research Centre, Riyadh, Saudi Arabia; ^4^King Saud University, College of Medicine, Riyadh, Saudi Arabia

## Abstract

*Background and Aim*. This is an open label prospective cohort study conducted at a tertiary care hospital. The primary endpoint is SVR12 in patients treated with sofosbuvir-based therapy in post-liver transplant patients with genotype 4 HCV recurrence.* Methodology*. Thirty-six treatment-experienced liver transplant patients with HCV recurrence received sofosbuvir and ribavirin ± peginterferon.* Results*. We report here safety and efficacy data on 36 patients who completed the follow-up period. Mean age was 56 years, and the cohort included 24 males and one patient had cirrhosis. Mean baseline HCV RNA was 6.2 log_10 _IU/mL. The majority of patients had ≥ stage 2 fibrosis. Twenty-eight patients were treated with pegylated interferon plus ribavirin in addition to sofosbuvir for 12 weeks and the remaining were treated with sofosbuvir plus ribavirin only for 24 weeks. By week 4, only four (11.1%) patients had detectable HCV RNA. Of the 36 patients, 2 (5.5%) relapsed and one died (2.75%).* Conclusion*. Our results suggest that sofosbuvir + ribavirin ± pegylated interferon can be utilized successfully to treat liver transplant patients with HCV recurrence.

## 1. Introduction

Hepatitis C virus (HCV) infection affects about 150 million individuals worldwide and is a major cause of chronic hepatitis C (CHC), hepatocellular carcinoma (HCC), and liver cirrhosis leading to liver transplant or death [1, 2]. Every year, 3-4 million people are infected with hepatitis C virus worldwide and more than 350,000 people die from hepatitis C related liver diseases [1]. After liver transplantation, recurrence of HCV infection occurs in almost all patients and is associated with reduced graft and patient survival [3–7]. However, survival of posttransplant patients who achieve sustained virological response (SVR) after treatment has been reported to improve compared to nontreated patients [8].

The overall prevalence of HCV in Saudi Arabia is estimated to be between 0.3 and 1.1% [9–12]. Hepatitis C genotype 4 is the most prevalent genotype in Saudi population, followed by genotype 1 [13].

Interferon used to be the only available treatment option in the past. However, this treatment option has its shortcomings as it has a complex and prolonged therapeutic course (24–48 weeks), substantial side effects, low barrier to resistance, and reduced efficacy in prior null responders or cirrhotic patients. In liver transplant setting, treatment of HCV recurrence with interferon and ribavirin has been reported to result in an SVR of only 30% [14–16].

Sofosbuvir is a new orally administered direct acting antiviral agent that inhibits HCV NS5B polymerase resulting in inhibition of HCV RNA replication [17]. Sofosbuvir is taken once daily at a dose of 400 mg. Multiple clinical studies have shown superiority of sofosbuvir-based therapy with high barrier to resistance when compared to the previous standard of care in both treatment-naive and treatment-experienced patients and across all HCV genotypes [18–25]. Because of its favorable safety profile and the lack of significant drug interactions compared to other agents, sofosbuvir has become the cornerstone in the management of HCV infection [26].

The accelerated disease progression in posttransplant setting with decompensation may reach rates as high as 40% annually [27]. Making a 5-year morbidity and mortality rates of posttransplant patients significantly higher than other HCV patients [28]. The use of sofosbuvir in post-liver transplant patients especially with genotype 4 has not been fully established. The aim of this study is to evaluate the safety and efficacy of sofosbuvir-based therapy in such population.

*Aims*. The aim of this study is to evaluate the safety and efficacy of the new polymerase inhibitor, sofosbuvir with ribavirin ± Peg-IFN in patients with recurrent HCV (genotype 4) infection, after liver transplant.

## 2. Methods 

### 2.1. Study Design

This is a prospective, interventional, single arm, open label study conducted at a major tertiary care hospital with large liver transplant program. Patients were enrolled in the study from January 2014 through March 2015. The study was approved by the local Institutional Review Board (IRB) (RAC#2141038). Informed consents were obtained from all patients.

Patients received sofosbuvir (Sovaldi®, Gilead Sciences Inc.) and ribavirin (Copegus®, Hoffmann-La Roche Ltd.), with or without pegylated interferon alfa-2a (Pegasys®, Hoffmann-La Roche Ltd.), for either 12 or 24 weeks.

### 2.2. Study Population

Post-liver transplant patients were eligible if they were ≥ 18 years, with recurrent HCV infection, genotype 4, and a positive HCV RNA and liver biopsy performed within one year prior to starting therapy with evidence of chronic HCV infection. All patients were treatment-experienced. HIV coinfected patients or those with known contraindications to sofosbuvir or RBV therapy and patients with GFR < 30 mL/min/1.73 m^2^ were excluded.

### 2.3. Study Endpoint

The primary endpoint of the study was undetectable HCV RNA 12 weeks after completion of therapy sustained virologic response 12 (SVR12). The secondary endpoint was to evaluate the safety and tolerability of sofosbuvir plus Peg-IFN plus RBV defined as development of any adverse event related to the study medication, as well as any episodes of acute or chronic rejection, during the follow-up period.

### 2.4. Efficacy Assessments

Serum HCV RNA levels were collected at baseline and weeks 4, 12, and/or 24. Serum HCV RNA detection and quantitation was performed using Abbott Real Time M2000 rt instrument. The HCV assay detects and quantitates genotypes [1–6]. The Abbott Real Time HCV assay provides detection limit (Analytical Measurement Range) from 30 to 100,000,000 IU/mL.

### 2.5. Safety Assessment

Vital signs were evaluated during patients clinic visit, electrocardiography and symptom-directed physical examinations were carried out when needed, and laboratory assessments were performed for biochemical analysis and hematologic testing. All side effects including those reported by patients were collected throughout treatment (during clinic visits) and until three months after completion of therapy. This includes any side effect that occurs upon initiation of therapy until 30 days after completion of therapy. An adverse effect was defined as any untoward medical occurrence secondary to any study medication, which does not necessarily have a causal relationship with this treatment.

### 2.6. Medication Dosages and Administration

Sofosbuvir was used at a dose of 400 mg tablet orally once daily as per the approved product labeling while Peg-IFN (pegylated interferon alfa-2a) was started at 180 mcg subcutaneously once weekly. Patients were not eligible for Peg-IFN if they had intolerance to IFN, had autoimmune hepatitis and other autoimmune disorders, had hypersensitivity to Peg-IFN or any of its components, or had decompensated hepatic disease, major uncontrolled depressive illness, a baseline neutrophil count below 1500/*μ*L, a baseline platelet count below 90,000/*μ*L or baseline hemoglobin below 10 g/dL, or history of preexisting cardiac disease

RBV (ribavirin) was dosed according to body weight (i.e., 1,000 mg for subjects weighing < 75 kg and 1200 mg for subjects weighing ≥ 75 kg) administered in two divided doses in accordance with standard practice or as per the treating hepatologist. We followed our institutional guidelines for immunosuppression management.

### 2.7. Statistical Analysis

Descriptive statistics for the continuous variables are reported as mean ± standard deviation and categorical variables are summarized as frequencies and percentages. Continuous variables were compared using Student's *t*-test while Chi-square test was used for categorical variables. The level of statistical significance is set at *p* ≤ 0.05. Intention-to-treat was used for safety analysis. For this study, all the statistical analysis was performed using the software package SAS version 9.4 (Statistical Analysis System, SAS Institute Inc., Cary, NC, USA).

### 2.8. Study Oversight

The study protocol was approved by the local Institutional Review Board (RAC# 2141038). This study was conducted in accordance with the latest version of the Declaration of Helsinki and Good Clinical Practice [29], the policies and procedures of Research Office Affairs (ORA) of the KFSHRC, and the national laws. All decisions regarding the patient medical management were solely made by the treating hepatologist according to his/her clinical judgment and based on international clinical practice guidelines whenever available. Only investigators had access to the study data.

## 3. Results

### 3.1. Study Patients and Disposition

Thirty-six patients were included in the study. Patients' baseline characteristics are presented in Table 1. We report efficacy data on 33 patients while the rest have not completed the follow-up period. All patients were treatment-experienced and all were on tacrolimus based immunosuppression. The median time from liver transplantation until starting therapy was 5.5 years (0.5–10 years). Thirty-three patients reached 12 weeks after the completion of therapy time point and are included in the analysis. Twenty-five patients received peg IFN + sofosbuvir + weight based ribavirin (group 1) and 11 patient received sofosbuvir and weight based ribavirin (group 2).

### 3.2. Efficacy/Virologic Response

Treatment with sofosbuvir and ribavirin resulted in rapid decline in HCV RNA levels in most patients. By week 4, only four patients (11.1%) had detectable HCV RNA (3 from the peg IFN-free group). Of the 33 patients included in the analysis, 30 patients (90.9%) achieved SVR12, 2 patients (5.5%) relapsed, and one died (2.75%) from HCC recurrence while on sofosbuvir therapy. Both patients who had relapsed were treated with sofosbuvir and ribavirin for 24 weeks. Among patients who did not achieve SVR12, one patient had detectable HCV RNA on week 4 (>30 m IU/mL). The remaining thirty-two patients had HCV RNA levels below the lower limit of quantification on week 4. The rest of the patients (3/36) have not completed the follow-up period; however, they all have achieved end of treatment response and were included in the safety analysis. See Table 2

### 3.3. Overall Safety

The most common adverse drug event (ADE) was anemia. Nine (25%) patients developed anemia requiring erythropoiesis-stimulating agent (ESA), of which 1 patient (2.7%) developed severe anemia requiring blood transfusion. Leukopenia requiring granulocyte colony-stimulating factor therapy developed in 1 patient (2.7%). There were no episodes of acute or chronic rejection, during the follow-up period. Other reported side effects included headache, fatigue, nausea, and muscle pain. None of these side effects required sofosbuvir therapy discontinuation. However, 1 patient discontinued peg IFN due to severe anemia and their treatment was extended to 24 weeks with sofosbuvir and ribavirin alone.

### 3.4. Immunosuppression Dosing

Tacrolimus levels were monitored according to the posttransplant local protocol. Mean plasma concentrations of tacrolimus were 5.5 ± 4.1 ng/mL with mean dose of 0.032 mg/kg per day at baseline and 4.5 ± 3.1 ng/mL at the end of treatment with mean dose of 0.05 mg/kg per day.

### 3.5. Dosing of Other Medications

*Ribavirin and Peg IFN*. The initial doses of ribavirin ranged from 1000 to 1200 mg daily (according to total body weight).

Overall, 10 patients (41.6%) had a modification in the ribavirin dose and 3 (18.7%) had modification in the peg IFN dosing during treatment due to the development of severe side effects (mainly anemia).

## 4. Discussions

In this study we report the safety and efficacy of sofosbuvir-based therapy in treatment-experienced patients with recurrent HCV (genotype 4) infection, after liver transplant. To our knowledge, this is one of very few studies evaluating the use of sofosbuvir-based therapy in such population.

There is limited data on the use of sofosbuvir in patients with HCV recurrence after liver transplant, especially with genotype 4. One case series reported three cases of HCV genotype 4 recurrence after liver transplant. All three patients were treatment-naive and were treated with simeprevir (SIM) and sofosbuvir (SOF) combination therapy for 12–24 weeks. All patients achieved SVR12 and therapy was well tolerated [30]. One study that included 40 patients (only one patient with genotype 4, and 88% were treatment-experienced) demonstrated that the administration of sofosbuvir plus ribavirin after liver transplantation in the setting of established HCV recurrence was well tolerated, and approximately 100% of patients had undetectable HCV RNA on treatment week 4 and at end of treatment. However, only 70% of the patients achieved SVR12 [31]. There were no cases of rejection or drug interaction, and there was no reported effect of sofosbuvir on serum levels of immunosuppressive medications, offering an all-oral therapy for treatment of HCV infection after liver transplantation [31]. The use of sofosbuvir plus ribavirin ± interferon in post-liver transplant patients has not been fully evaluated [32]. We reported real life experience utilizing a sofosbuvir-based therapy after liver transplant in patients with mainly genotype 4 HCV recurrence.

Several studies have shown that graft and patient survival are lower among liver transplant recipients with HCV infection than patients with other etiologies, owing to accelerated disease progression following graft reinfection. Unlike telaprevir and boceprevir, there is no significant interaction between sofosbuvir and immunosuppressive medication. In our study, doses of calcineurin inhibitors were slightly modified during the treatment period according to drug levels which is consistent with previously reported studies [32]. The rates of serious adverse events and study drug discontinuations due to adverse events are higher in other studies with utilizing interferon-free regimens compared to ours [33].

Few studies have looked at the performance of sofosbuvir in real life clinical practice in post-liver transplant population. Forns et al. described a series of 84 liver transplant patients (all genotypes), treated with sofosbuvir plus ribavirin ± Peg-IFN. In that study, half of the patients had compensated or decompensated post-liver transplant cirrhosis, while the remaining had severe cholestatic hepatitis C or early HCV related disease recurrence (Metavir F2-F3) and only 3 patients had genotype 4. Overall 30% of patients stopped treatment, 12.5% died, and 11.5% discontinued the drug due to adverse events and 6.7% due to retransplantation. Among those who completed therapy, 70% improved, 13% remained stable, and 17% worsened or died due to disease progression. The end of treatment response was 87% with an SVR12 of 62% [34]. Another study, by Satokar et al. [35], looked at three direct acting antiviral-based regimens for HCV treatment after liver transplant including sofosbuvir plus simeprevir: the second group was sofosbuvir plus ribavirin and the third group was sofosbuvir plus ribavirin plus peg IFN for a total of 59 patients. HCV genotypes 1, 2, 3, and 4 were present in 29, 3, 25, and 2 patients, respectively. Although more patients in the group who received triple therapy had undetectable HCV RNA by week 4 of therapy, the results were clinically significant despite not achieving statistical significance; 66.7% in the triple therapy group (i.e., sofosbuvir plus ribavirin plus peg IFN) had undetectable HCV RNA by week 4 of therapy versus 42.9% in those who received sofosbuvir plus RBV alone [36]. This may suggest a potential added benefit of peg IFN inclusion to the regimen. As a matter of fact, patients who received IFN in addition to sofosbuvir and ribavirin had the highest percentage of undetectable HCV RNA by week 4 of therapy compared to the other treatment groups [35].

Another study looked at the treatment of HCV recurrence after liver transplant using sofosbuvir plus daclatasvir ± ribavirin and therapy was well tolerated with rapid decline in HCV RNA levels [37]. A study in post-liver transplant patients including patients with fibrosing cholestatic hepatitis reported a liver transplant recipient with severe cholestatic HCV who was successfully treated with the sofosbuvir and daclatasvir with very good safety and efficacy [38, 39]; none of our patients in our cohort was treated for FCH.

The rapid decline in HCV RNA with direct acting antiviral therapy, including sofosbuvir, has been modeled using a multiscale age-structured approach [40, 41], which may indicate a triphasic pattern of serum viral load decline. The model suggests that 6–8 weeks of suppression of HCV RNA (continuously undetectable) is required for complete virologic clearance. The magnitude of HCV RNA decline in these patients is also similar to that observed with sofosbuvir in phase 3 studies, reflecting the enhanced rates of loss of intracellular viral RNA, replication templates, and infected cells.

The optimal tactics to prevent HCV recurrence after transplant including treatment of HCV infection before transplant have been largely discussed [42, 43]. One study demonstrated that graft reinfection can be significantly reduced if patients are treated with sofosbuvir containing regimen while awaiting transplant and had undetectable HCV RNA for at least one month prior to transplant [42, 44]. Sofosbuvir has multiple potential advantages when compared to first generation direct acting antivirals (DAA). In addition to higher SVR rates, the drug-drug interaction profile with other medications and especially with calcineurin inhibitors (CNIs) is of particular importance. Multiple studies have shown lack of significant interactions with CNIs that may require any dose adjustment [45]. These data came from studies in patients after liver and kidney transplant [46] which were consistent with the findings of our study.

Another combination that has been safely used in post-liver transplant patients is sofosbuvir plus simeprevir in patients with significant fibrosis [47, 48]. Another combination that has been recently approved by US FDA is sofosbuvir plus ledipasvir (Harvoni®) which has also been studied in genotype 4 population. The safety and efficacy in post-liver transplant patients have also been established [43, 44].

Extrapolation of these results to all patients with HCV recurrence after liver transplant might not overestimate the SVR12 rates due to the fact that the population studied is composed of patients with moderate to severe fibrosis who are treatment-experienced with relatively high viral load at baseline. Thus, in other easy-to-treat populations, SVR12 rates are expected to be higher. In conclusion, therapy with sofosbuvir and ribavirin ± INF after liver transplantation in treatment-experienced patients resulted in 83.3% SVR12. Given the burden of disease, the increased morbidity, mortality, and costs, these results provide hope for patients in need.

## Figures and Tables

**Table 1:** Patients' baseline characteristics.

	Number total (36)	Percent (%)
Gender		
Female	12	33.3%
Male	24	66.6%
Age, years (mean ± SD) Range	56.3 (±9.8) 27–70	
Time from transplant (years)	5.5	
Comorbidities		
Diabetes	9	25
Hypertension	10	27.7
Dyslipidemia	8	22.2
Others	15	41.6
Indication of liver transplant		
HCV-HBV-HCC	1	2.7
HCV	30	83.3
HCV-AIH	1	2.7
HCV-HCC	4	11.1
Donor type		
Living	5	
Deceased donor	31	
HCV RNA at baseline (IU/mL)	8,011,643.0 (±20,301,887.4)	
Liver enzymes at baseline (unit/liter)		
ALT	69.7 (±59.2)	
AST	67.3 (±47.2)	
ALP	181.2 (±139.4)	
GGT	422.2 (±612)	
Primary immunosuppressive medication		
Tacrolimus (FK)	36	100
Cyclosporine (CSA)	0	0
Other medications		
Prednisone	0	
Mycophenolate mofetil	9	25
Biopsy staging:		
0	2	
1	3	
2	13	
3	9	
4	1	
Unknown	5	
Regimen		
Sofo + peg IFN + RBV for 12 weeks	28	77.7
Sofo + RBV for 24 weeks	8	22.33

Plus-minus values are means ± SD. HCV: hepatitis C virus; HBV: hepatitis C virus; HCC: hepatocellular carcinoma; AIH: autoimmune hepatitis; ALT: alanine aminotransferase; AST: aspartate aminotransferase; ALP: alkaline phosphatase; GGT: gamma-glutamyl transpeptidase. The Metavir fibrosis score (on a scale from F0 to F4, with F0 indicating no fibrosis and F4 fibrosis consistent with compensated cirrhosis) was based on a liver-biopsy specimen reviewed by a central pathologist.

**Table 2:** Virologic response during and after treatment.

	Regimen 1 (25) Sofosbuvir + ribavirin + peg IFN for 12 weeks	Regimen 2 (11) Sofosbuvir + ribavirin for 24 weeks
Mean HCV PCR baseline (IU/mL)	5,377,975	13,897,828
During treatment		
Week 4		
HCV RNA < 30 IU/mL	25 (100%)	9 (81%)
End of treatment		
HCV RNA undetectable	25 (100%)	11 (100%)
After treatment		
Week 12	(25/25) 100%	(6/9) 75%^∧^

^∧^Three patients did not complete follow-up period yet.

Two patients relapsed and one deceased while on sofosbuvir therapy.

Virologic relapse was defined as a confirmed HCV RNA level of 25 IU per milliliter or more between the final visit and 12 weeks after the last dose of study drugs among patients who had an HCV RNA level of less than lower limit of quantification (LLOQ) at the final visit.

## Abbreviations

AASLD: American Association of the Study of Liver Disease
BMI: Body mass index
CHC: Chronic hepatitis C
CPT: Child-Turcotte-Pugh score
CSA: Cyclosporine
DAA: Direct acting antiviral
DAC: Daclatasvir
EASL: European Association for the Study of Liver Disease
FK: Tacrolimus
GT1: Genotype 1
GT2: Genotype 2
GT3: Genotype 3
GT4: Genotype 4
GT5: Genotype 5
GT6: Genotype 6
HBV: Hepatitis B virus
HIV: Human immunodeficiency virus
LED: Ledipasvir
LLOQ: Lower limit of quantitation
MELD: Model for end-stage liver disease
MMF: Mycophenolate
Peg IFN: Pegylated interferon
RBV: Ribavirin
SIM: Simeprevir
SOFO: Sofosbuvir.
